# Clinical Features of Varicella-Zoster Virus Infection

**DOI:** 10.3390/v10110609

**Published:** 2018-11-02

**Authors:** Peter G. E. Kennedy, Anne A. Gershon

**Affiliations:** 1 Institute of Infection, Immunity and Inflammation, College of Medical, Veterinary and Life Sciences, University of Glasgow, Garscube Campus, Glasgow G61 1QH, Scotland, UK; 2 Department of Pediatrics, Columbia University College of Physicians and Surgeons, New York, NY 10032, USA; aag1@cumc.columbia.edu

**Keywords:** varicella, herpes zoster, VZV, infection, postherpetic neuralgia, enteric zoster, neurology, acyclovir, valacyclovir

## Abstract

Varicella-zoster virus (VZV) is a pathogenic human herpes virus that causes varicella (chickenpox) as a primary infection, following which it becomes latent in peripheral ganglia. Decades later, the virus may reactivate either spontaneously or after a number of triggering factors to cause herpes zoster (shingles). Varicella and its complications are more severe in the immunosuppressed. The most frequent and important complication of VZV reactivation is postherpetic neuralgia, the cause of which is unknown and for which treatment is usually ineffective. Reactivation of VZV may also cause a wide variety of neurological syndromes, the most significant of which is a vasculitis, which is treated with corticosteroids and the antiviral drug acyclovir. Other VZV reactivation complications include an encephalitis, segmental motor weakness and myelopathy, cranial neuropathies, Guillain–Barré syndrome, enteric features, and zoster sine herpete, in which the viral reactivation occurs in the absence of the characteristic dermatomally distributed vesicular rash of herpes zoster. There has also been a recent association of VZV with giant cell arteritis and this interesting finding needs further corroboration. Vaccination is now available for the prevention of both varicella in children and herpes zoster in older individuals.

## 1. Introduction

Varicella-zoster virus (VZV) is a pathogenic human alpha-herpesvirus that causes chickenpox (varicella) as a primary infection, which usually occurs in children in locales where vaccination is not practiced [1]. Following the primary infection, this neurotropic virus becomes latent, primarily in neurons in peripheral autonomic ganglia throughout the entire neuroaxis including dorsal root ganglia (DRG), cranial nerve ganglia such as the trigeminal ganglia (TG), and autonomic ganglia including those in the enteric nervous system [1,2,3]. Up to decades later, latent VZV may reactivate, either spontaneously or following one or more of a variety of triggering factors to cause herpes zoster (shingles), which usually appears as a painful or pruritic cutaneous vesicular eruption that occurs in a characteristic dermatomal distribution [1,2]. This viral reactivation becomes more frequent with the increased age of the human host because of diminished cell-mediated immunity to the virus in such individuals [4,5]. Other specific triggers for viral reactivation include immunosuppression from disease or drugs, trauma, X-ray irradiation, infection, and malignancy [1]. While the main and most important complication of herpes zoster is postherpetic neuralgia (PHN), it has been increasingly recognised over the last decade that VZV reactivation causes a variety of acute, subacute, and chronic neurological syndromes, so its clinical manifestations are protean [3].

VZV is a double-stranded DNA virus with a genome of just under 125,000 base pairs and it contains 68 unique open reading frames (ORF) [6]. The mechanisms of VZV latency are slowly being unravelled, but several issues remain to be clarified. It is known that during ganglionic latency, VZV DNA is located predominantly, if not exclusively, in neurons [7] in which it is present in a nonintegrated form, probably as endless episomes of unit or concatemeric length [8,9]. It has been known for some time that viral transcription during latency is highly restricted, with transcripts for VZV gene 63 being the most commonly detected transcript [10,11,12], and previous work using different techniques has also reported transcription of VZV genes 21, 29, 62, and 66 [10,11,12,13]. However, a problem with many previous reports is that the ganglia obtained at autopsy have been studied only after 12–48 h after death, at which time the process of viral reactivation may well have already started. Indeed, when human ganglia were analysed at less than 9 h after death, no transcripts for VZV were detected, though VZV ORF63 transcript levels in human TG increased with longer postmortem intervals [14]. This study suggested that expression of other VZV genes previously detected was probably a reflection of viral reactivation, a view that is supported by the detection by multiplex polymerase chain reaction (PCR) of several VZV ORFs, including those other than those just corresponding to immediate-early or early transcripts [15]. On the other hand, studies of human enteric ganglia removed during gastrointestinal surgery from children immune to varicella and placed immediately in “RNA later” solution revealed transcripts for ORFs 63, 4, and 66 [16]. One possibility is that when analysing human ganglia for VZV latency, both true latent transcripts and those indicating a degree of low-level viral reactivation are being detected unless the ganglia are studied prior to 9 h postmortem. Very recently, a unique spliced latency-associated VZV transcript was detected in human TG neurons which maps antisense to the viral transactivator gene 61 [17]. Since the latter ganglia studied had been obtained at about 6 h after death, it is clear that this could not have been detected due to viral reactivation. Given the inconsistent results in various laboratories, the molecular status of VZV during latency needs further study.

In this review, we consider the main clinical manifestations of VZV reactivation, the most common of which is generally recognised as being herpes zoster, which may be followed by PHN. Further, a wide variety of neurological features may also be caused by VZV reactivation from latency and these are mentioned here. We also review the current evidence for the benefit of VZV vaccination in both children and adults.

## 2. Varicella and Its Complications

The primary infection with VZV is varicella, commonly known as chickenpox. Varicella is highly contagious; it is most commonly seen in children under the age of 10 years in countries where live attenuated varicella vaccine is not routinely administered [1]. The major feature of the illness is a vesicular pruritic rash that occurs mainly on the trunk, head, and face. The extremities are somewhat spared; skin vesicles are full of infectious, well-formed virus which are aerosolized and serve to transmit VZV to others who have not had the disease previously. The skin lesions commonly occur in crops and progress from papules to vesicles to crusts over a few days. There may be anywhere from a few to many hundreds of vesicles, with an average of about 500. More severe cases manifest more severe rashes and take longer to heal. Concomitant symptoms include malaise, fever, and fatigue, and the illness usually lasts about a week. Complications include bacterial superinfection of the skin, encephalitis, and pneumonia. Adults and immunocompromised patients are more prone to severe infections than healthy children [1,18,19,20].

Individuals who have received live attenuated varicella vaccine may still develop varicella after an exposure to the virus (either a person with varicella or one with zoster). Patients with zoster can transmit varicella to others but with a lower attack rate of chickenpox than those with primary infection [20]. Vaccinees who nevertheless develop varicella usually have mild cases with fewer vesicles and complications. This situation is termed “breakthrough varicella” and is less contagious than primary varicella. Vaccinees who have had only one dose of vaccine are much more likely to transmit the virus to varicella susceptibles who are exposed than those who have had two doses of vaccine. When a person develops varicella despite receiving two doses of vaccine, the disease is often very minor and may be difficult to diagnose as varicella both clinically and in the laboratory [1,20].

The diagnosis of VZV infection is usually made clinically by the appearance of the skin rash. In confusing or unusual appearing cases, the diagnosis may be made by identifying VZV DNA in skin lesions by PCR. Culture of VZV from skin lesions may also be used, but it is more expensive, takes more time, is poorly available, and is less sensitive than PCR [20]. In patients with suspected meningitis or encephalitis and other complications due to VZV, the viral DNA may be demonstrable in cerebrospinal fluid and/or saliva [18,20].

The infants of women with varicella in the first 20 weeks of pregnancy are at about a 2% risk of developing the congenital varicella syndrome [1]. These infants often have a variety of severe abnormalities of their brain, eyes, extremities, and skin and most succumb in infancy or early childhood. They frequently experience recurrent VZV reactivation and may have multiple cases of clinical zoster [20]. Fortunately the syndrome is unusual in that only about 2% of women who develop varicella in pregnancy give birth to an infant with the congenital varicella syndrome [20].

Adults are more likely to experience severe varicella than children. Severe and even fatal varicella, moreover, often occurs in patients who are immunocompromised due to disease or medications such as corticosteroids or cancer chemotherapy [1,20]. These patients manifest extensive, often haemorrhagic rashes and may also develop complications such as pneumonia, hepatitis, and/or encephalitis all due to VZV. They are likely more prone to develop severe bacterial infections as well. Severe varicella may be prevented to some extent by administration of passive immunization with VariZig, a form of immunoglobulin containing high titers of antibodies to VZV; passive immunization should be administered as soon as possible after a recognised close exposure to VZV in a high-risk person who has never had varicella [20].

Routine treatment of varicella in otherwise healthy children is not uniformly recommended, although an oral form of the antiviral acyclovir is available. Otherwise, healthy adults and immunocompromised patients who develop varicella should receive treatment. If it develops or seems to be developing, severe varicella should be treated with intravenous acyclovir. For the best outcome, antivirals should be given as soon as possible to immunocompromised individuals and to anyone who seems to be developing severe varicella [20].

Patients who develop zoster should be treated as soon as possible with acyclovir, famciclovir, or valacyclovir, which are administered orally. If zoster is severe, especially in immunocompromised patients, intravenous acyclovir can be administered, especially at the start of treatment [20]. Although generally well tolerated, adverse effects of antiviral therapy for which clinical monitoring is necessary include gastrointestinal, neurological, and renal toxicity [1,21].

An important feature of varicella is the development of a viremia that just precedes the appearance of the rash. The virus is carried to the skin in T lymphocytes, where the rash develops [21]. Latency in DRG, Cranial nerve ganglia CNG, and also to autonomic ganglia may be established by two mechanisms during the viremia and as VZV travels directly from skin to ganglia by anterograde transport to DRG and CNG [1,21].

## 3. Herpes Zoster

Reactivation of VZV in neurons occurs with unknown frequency but is possibly very common [22]. Over 50 years ago, Hope Simpson postulated that reactivation was frequent and could occur with or without symptoms [22]. The reality of subclinical reactivation was demonstrated when it was determined that one-third of astronauts developed reactivation of VZV transiently during space travel [23]. The diagnosis was made by finding VZV DNA in saliva; the astronauts had no symptoms of zoster and the viral DNA disappeared within a few weeks after return to Earth [23]. Importantly, it is very rare to isolate infectious VZV from saliva of patients with active or subclinical VZV infections [24].

When symptomatic reactivation of VZV occurs, the condition is termed herpes zoster, often referred to as “zoster”. Although it is now recognised that zoster may occur in the absence of rash, the classical presentation is appearance of a unilateral, dermatomal rash that is painful, pruritic, or both. The causes and mechanisms of reactivation remain unclear, but zoster is associated with a preceding decrease of cellular immunity (CMI) to VZV [4,5]. Vaccination against zoster is aimed at restoring CMI to VZV to prevent zoster from occurring [1].

Since the process of reactivation is not fully understood, the incubation period of zoster is unknown. Characteristically, zoster presents with a unilateral vesicular rash on the face, head, or trunk, although it can also occur on the extremities. The vesicles are full of infectious virions that can become airborne and infect nearby varicella susceptibles as chickenpox, although zoster is only about half as contagious as varicella. The zoster rash may be mild and heal quickly or it can be severe with extensive lesions that may last for weeks. The latter possibility is more likely to occur in elderly patients or immunocompromised individuals than others [1].

### Postherpetic Neuralgia (PHN)

Patients who develop PHN are often those who have experienced zoster with many skin vesicles accompanied by severe pain. At some point after resolution of these symptoms, usually within about three months, the persistent pain of PHN begins in the area of the healed rash. The pain ranges from mild to extremely severe and may be very debilitating, especially in the elderly. It may last for a year or longer. Pain is often described as throbbing, burning, or shooting. A prominent symptom is allodynia, in which a mere touch of the skin even by light clothing causes intense pain. The mechanism by which PHN occurs is not entirely understood [1,20]. Two leading theories are that the excitability of ganglionic neurons is altered and/or that there is a form of persistent VZV infection (not latency) in involved ganglia [25]. Unblinded studies have described improvement in PHN with antivirals administered for months [26], but definitive information awaits the performance of a double-blind study of antiviral therapy vs. placebo. Vaccines aimed at preventing zoster (see below) are useful to prevent PHN from occurring. Although it is possible that early antiviral therapy in zoster may dampen the character of PHN that follows, there is currently general agreement that antivirals should not be used to treat established PHN [27]. There is no known cure for PHN, although gabapentin and some antidepressive medications have been tried with some success. Patients with severe PHN should be referred to a pain specialist.

## 4. Neurological Complications of VZV Reactivation

It is now recognised that VZV reactivation is associated with a wide variety of neurological complications. When there is a long delay between the VZV episode and the neurological condition, it may be difficult to prove a clear causal relationship, but this usually seems logical in the absence of an alternative explanation. The key complications are now outlined, but it should be pointed out that for many conditions, our knowledge is based on a relatively small number of reported cases, though for some, such as vasculopathy, the association is strong.

### 4.1. VZV Vasculopathy

Probably the most important neurological complication of VZV reactivation is a vasculopathy due to a productive viral infection of both large and small cerebral arteries, though its exact frequency is unknown [19]. However, since recent evidence has established that herpes zoster infection is a risk factor for stroke, and since zoster itself is frequent, occurring in about half of all individuals by the age of 85 years [19], this suggests that VZV vasculopathy is probably not an uncommon complication. The clinical presentation may be highly variable including both ischaemic and haemorrhagic stroke, cerebral aneurysm, temporal artery involvement (which is discussed below), arterial dissection, transient ischaemic attacks, cerebral venous thrombosis, and spinal and peripheral artery thrombosis [19,28,29]. Pathologically, an inflammatory infiltrate with T cells and macrophages is seen in the adventitia and intima of the affected arteries and also the media at a later stage [19]. Infected arteries usually contain multinucleated giant cells, Cowdry A inclusion bodies, herpes virions, and VZV DNA and antigens [30,31]. The clinical presentation of VZV vasculopathy varies considerably, but a typical case may present weeks or months after the zoster rash or even without a rash (see below), followed by neurological features such as progressive cognitive impairment, seizures, and other focal signs [3,19]. Both MRI and CT may show evidence of ischaemia or haemorrhage and angiography may suggest a vasculitis. The diagnosis may be established by the detection by PCR of VZV DNA in the cerebrospinal fluid (CSF) of the affected patient, though it has been shown that the presence of anti-VZV IgG antibody in the CSF can also establish the diagnosis and is more sensitive than PCR for DNA, which may sometimes be negative in VZV vasculopathy [19,28]. Treatment should be with a two-week course of intravenous acyclovir and a one-week course of oral corticosteroids, though in the case of immunocompromised patients and relapsing cases, the duration of corticosteroid and antiviral therapy should be longer than in the immunocompetent [28].

### 4.2. Giant Cell Arteritis and VZV

In view of the ability of VZV to cause a vasculopathy, it was reasonable to investigate whether VZV might also play a pathogenetic role in giant cell arteritis (GCA), in which there are inflammatory changes in the temporal arteries (TAs) and a serious risk of sudden blindness. Gilden and colleagues [30] examined TA sections from a total of 82 biopsied cases of GCA and detected VZV antigens in 74% of GCA positive TAs, mainly in “skip” regions, but were positive only in 8% of normal TAs and 38% of skeletal muscle sections adjacent to the VZV antigen positive areas. Subsequently, this group also analysed GCA negative but clinically positive TAs and found that 64% of these and also 22% of normal TAs contained VZV antigens [31]. They concluded from these studies that, irrespective of whether or not they show characteristic histopathology, TAs from patients with clinically suspected GCA contain VZV antigens. If VZV is truly causing at least a proportion of GCA, then it follows that GCA patients with demonstrable VZV in their TAs should be treated with both corticosteroids and acyclovir. However, a recent study [32] using the same techniques only detected VZV antigens in only 3/25 (12%) of TAs in sections of biopsy-proven GCA. They also found that false positive staining for VZV antigens was detected in several TA biopsy sections. The problems of false-positive staining of human tissue sections with antibodies to VZV due to antibody cross-reactivity were also emphasised in another recent study [33], where caution was advised in interpreting such apparently positive staining. As well as the problems of possible nonspecific staining, the presence of VZV antigens in some normal TAs, and the varying positive detection rates in different studies, a critical issue is the one of a cause-and-effect relationship between the viral detection and the production of the human disease [34]. Gilden [30] considered a causal relation likely and that there may be a subgroup of patients with GCA in which VZV is a critical determinant in whom corticosteroids alone in the absence of antiviral therapy may actually be deleterious since it may allow an ongoing untreated viral infection to persist. It seems likely that the only definitive way of proving a causal effect of VZV in GCA is to carry out a prospective clinical trial of corticosteroids alone vs. corticosteroids plus acyclovir in biopsy-proven GCA to determine whether the former has a substantial benefit. A recent study [35] which did not find a decreased incidence of GCA in individuals who had received VZV vaccination is certainly noteworthy but does not by itself disprove a relation between VZV reactivation and GCA. At present, the situation remains somewhat unclear and further studies need to be carried out to confirm or refute these remarkable findings of VZV in TA.

### 4.3. Segmental Weakness and Myelopathy

Segmental motor weakness occurring after an episode of herpes zoster is well recognised, though the interval between the rash and the weakness may be highly variable from a day to several months [28]. While the rash and weakness usually occur in the same region, there may be a topographical dissociation between the two in about 10% of cases [28]. The weakness may affect the upper or lower limbs, the diaphragm, intercostal muscles, or the sphincters [28]. The structures involved in zoster-associated weakness may be the anterior or posterior spinal roots, the anterior or posterior spinal horns, or the brachial plexus, findings that may be confirmed by MRI and/or electrophysiological investigations [36]. The prognosis is generally thought to be quite favourable, with complete recovery occurring in about 55–75% of cases [28,36], though these figures may be somewhat optimistic in the authors’ experience. Treatment when the diagnosis is certain should be with a 14-day course of intravenous acyclovir and a 5–7-day course of oral corticosteroids. Related to this, muscle and sphincter weakness may also be caused by a zoster-associated myelitis in which the spinal cord is preferentially affected by the virus. The myelopathy thus produced may be acute or chronic, usually occurs one to two weeks after the rash, is symmetrical with a typical bilateral leg, and also presents sphincter weakness. The diagnosis is usually established by the prior herpes zoster, the clinical pattern, and using investigations such as MRI of the relevant region of the spinal cord which may show characteristic T2 hyperintense lesions and cord swelling as well as a typical CSF pleocytosis and the demonstration by PCR of VZV DNA and/or anti-VZV IgG antibody in the CSF [28]. Treatment is the same as for VZV-associated myelitis.

### 4.4. VZV Encephalitis

A meningoencephalitis has also been described as occurring in association with herpes zoster, though it may precede, be simultaneous with, or occur after the rash itself. This complication is comparatively rare and may be mild (and therefore under-reported), and in the opinion of Gilden, some cases of VZV encephalitis may actually be a vasculopathy [36]. However, in the authors’ opinion, an encephalitis in the absence of a vasculopathy may rarely occur in association with zoster and may complicate around 0.25% of all zoster cases. The pathogenesis is not understood. The clinical picture is with a relatively mild onset, either acute or more gradual, or an encephalomyelitis with headache, fever, and neck stiffness if there is an associated meningitic element. The illness may be more serious in immunocompromised individuals such as those with AIDS. During the illness, there may also be motor weakness. There is a CSF pleocytosis and the diagnosis may be established using PCR to detect VZV DNA in the CSF, which may also contain anti-VZV IgG antibody. The EEG (electroencephalogram) may be normal or else show nonspecific abnormalities. Treatment should be with a 14-day course of intravenous acyclovir and possibly also a one-week course of oral corticosteroids.

### 4.5. VZV Cranial Neuropathies

The cranial nerve that is most frequently described as affected [36] by herpes zoster is the seventh cranial nerve, also known as the facial nerve. When this occurs, it is called the Ramsay Hunt syndrome, which classically is the combination of otic zoster and ipsilateral facial paralysis as described by Hunt in 1907. The cause is thought to be herpes zoster affecting the geniculate ganglion. Clinically, the syndrome produces otalgia with tinnitus, deafness and vertigo, zoster vesicles in the external auditory meatus, and loss of taste in the anterior two-thirds of the tongue due to involvement of the chorda tympani branch of the facial nerve [36]. This syndrome may be associated with dysfunction of other cranial nerves as well as a localised brainstem encephalitis. Treatment is with a combination of oral acyclovir (or valacyclovir or famcyclovir) and corticosteroids.

When zoster affects the ophthalmic division of the trigeminal (fifth) cranial nerve, there may follow a number of local ocular complications such as keratitis, scleritis, iritis, and retinitis, which require specialist ophthalmic attention. Further, ophthalmoplegia may occur in a small proportion of cases of cephalic zoster Most of the cranial nerves have been described at some stage as being affected by herpes zoster, either alone or together with others [36].

### 4.6. VZV and Guillain–Barré Syndrome (GBS)

Although GBS is a well-recognised complication of varicella [37], this neurological syndrome is thought to only rarely follow an episode of herpes zoster. The paucity of published cases, together with the fact that both GBS and herpes zoster are relatively common diseases, makes the attribution of a definite association between the two conditions somewhat problematic as it might be fortuitous. However, zoster does genuinely appear to be occasionally followed by GBS where the clinical picture is similar to the disease described due to other triggering factors [37]. However, the prognosis in zoster-associated GBS appears to be poorer than other cases, and a shorter latent period between the rash and the GBS has been reported to be associated with a worse outcome compared to cases occurring after a longer (>2 weeks) latent interval [37]. Treatment should be the same as is given in other cases of GBS and the pathogenesis is not understood, though an immune-mediated mechanism seems the most likely.

### 4.7. Zoster Sine Herpete

Zoster sine herpete (ZSH) is the term used when typical dermatomal pain due to VZV occurs in the absence of the characteristic rash. That VZV is indeed the cause of such symptoms was proved by Gilden and his colleagues by demonstrating the presence of VZV DNA by PCR in the CSF of two such patients in whom treatment with acyclovir improved their dermatomal pain [38]. The diagnosis of ZSH can be established by PCR detection of VZV DNA in the CSF or peripheral blood mononuclear cells or by the presence of anti-VZV IgG antibody in the CSF [3,39]. It is possible to make a diagnosis of ZSH by virtue of the presence of anti-VZV IgG antibody even when no VZV DNA can be detected. It has emerged in recent years that the spectrum of ZSH is much wider than was previously thought. Indeed, VZV vasculopathy often presents in the absence of a preceding rash [39]. While this spectrum of syndromes is increasing, it is important that the clinician suspects a diagnosis of ZSH in any patient with persistent radicular pain or who presents with apparently undiagnosed acute, subacute, or chronic cerebral or spinal cord features, especially in the absence of a rash and the presence of a CSF pleocytosis [39].

## 5. VZV and Enteric Complications

The development of an in vitro model of VZV infection with features of latency and reactivation in guinea pig enteric neurons led to the search for whether latent VZV could be found in human enteric neurons [40]. Studies of children undergoing routine gastrointestinal (GI) surgery led to the identification of latent GI VZV by demonstrating ORFs 63, 4, and 66 in enteric neurons (typical of latent infection) but no transcripts from lytic genes such as ORF 68 [16]. Subsequent identification of VZV DNA in saliva from children and adults with zoster led to a means to detect reactivation of VZV in the GI tract in the absence of skin lesions [18]. Stomach and colonic ulcers caused by VZV were identified by VZV in saliva and by immunofluorescent assays on GI tissues [18,41]. Further studies on enteric zoster are being conducted. Current results indicate that these conditions are not rare. Presumably, VZV reaches enteric ganglia during the viremia of varicella and establishes latency there. Latent vaccine virus (Oka strain) has also been demonstrated on occasion [18,41]. It is possible that asymptomatic reactivation of VZV in the GI tract, which is the largest immune organ in the body, plays some role in maintaining long-term immunity to the virus.

## 6. VZV Vaccinations

Development of a live attenuated vaccine to prevent varicella was accomplished by Takahashi in 1974 [42]. Initially, use of the vaccine was controversial. There was no doubt that it was important to prevent severe varicella, especially in immunocompromised patients; at that time, it was the early days for antiviral therapy. Whether it was safe, however, to immunize children with a live virus that caused latent infection that could reactivate was problematic for many [20]. It did not take long, however, for it to be demonstrated that varicella vaccine could decrease morbidity and mortality and was safe to administer to children with leukemia in remission [43].

Given the success in immunocompromised children, it was then decided to conduct clinical trials in healthy children who were susceptible to varicella in the United States. These trials too were highly successful, and live attenuated varicella vaccine was eventually licensed in the United States for all healthy children, eventually in a two-dose regimen [20]. Today, varicella vaccine is used all over the world, although many countries are not yet using a two-dose schedule. One dose offers about 85% protection, while two doses protect about 98% of vaccinees [44,45]. Widespread immunization of healthy infants and children has led to herd immunity in the United States, where varicella vaccine is no longer offered to immunocompromised patients. Varicella has now become rare in the United States; there has been a dramatic concomitant fall in hospitalizations and deaths (87% decrease) from varicella as well [44,45,46].

Only a few countries refuse to immunize children routinely; among them is England, where modeling studies have suggested, but far from proven, that widespread varicella immunization results in less circulation of wild-type VZV, leading to an increase in zoster in middle-aged persons. England, therefore, still has children who die of varicella every year. A controversy as to whether varicella vaccine increases the incidence of zoster due to less boosting of immunity has grown up. Most investigators reject the idea that varicella vaccination increases zoster incidence [47]. For one thing, while the incidence of zoster is increasing in the United States, this increase began in the 1950s, long before the varicella vaccine was developed. Zoster is also increasing in countries where the varicella vaccine is not being used, and the increase is probably multifactorial and includes increased identification of zoster, an aging population, and more and more immunocompromised people in the population, including those on biologicals to control autoimmune and other diseases [20,44].

The availability of live attenuated varicella vaccine led directly to the development of a live vaccine to prevent zoster. In order to boost cellular immunity in older individuals who had varicella many years ago, it was necessary to use a formulation of vaccine 14 times as strong as varicella vaccine. This vaccine, known as Zostavax^TM^ and produced by Merck and Co., led to protection against zoster and PHN in 50–60% of individuals over 60 years of age [48]. This protection, unfortunately, begins to wane, however, in some cases as early as the first year after immunization and is essentially gone within eight years [49]. Boosters are not recommended. This vaccine is not guaranteed safe for immunocompromised persons, in whom it may cause serious VZV infections [50].

In order to try to develop a vaccine that would provide better protection of older individuals and be safe for vaccination of immunocompromised patients, a new vaccine, Shingrix^TM^, was developed by Glaxo Smith Kline. This vaccine is a “subunit” vaccine containing as the antigen the main glycoprotein of VZV, termed “glycoprotein E”, along with an adjuvant ASO1B that enhances innate and adaptive cellular immunity to VZV. This vaccine requires two doses, given two to six months apart. It provides about 97% protection to healthy persons as old 70 years of age when immunized. It also provides protection against PHN, which is notoriously difficult to treat. It is currently being tested for safety and immunogenicity in immunocompromised patients [51,52]. The most challenging aspect of Shingrix^TM^ is that it results in a high incidence of side effects for the first few days after immunization, including reactions at the injection site, fever, and malaise. Relatively serious adverse effects, however, are rare [51,52].
